# Could global surgery overcome a decline in surgical cases?

**DOI:** 10.1016/j.amsu.2022.103704

**Published:** 2022-05-02

**Authors:** Robert B. Laverty, Rahul M. Jindal

**Affiliations:** a Brooke Army Medical Center, San Antonio, TX, USA; b Uniformed Services University, Bethesda, USA

**Keywords:** Global surgery, Surgical volume, Minimal invasive surgery, COVID 19, Reduction duty hours, ACGME, Accreditation Council for Graduate Medical Education, DHR, duty-hour restrictions, LMIC, low and middle income countries, HIC, high income countries

## Abstract

• There have been three distinct landmarks for the US surgical trainees leading to a decline in surgical volume and in open number of cases.
• Global surgery experiences have been adopted to expose trainees to surgical problems not routinely seen in the Global North.
• Global Surgery also exposes trainees to empathic and collaborative approaches.
• Benefits of global surgery to compensate for the decline in volume, variety and open surgical cases need to be studied through an academic, ethical, and economic lens.
• LMICs trainees could travel to HIC for research and clinical training in exchange for the skills and case volume that HIC trainees would obtain in LMICs.

General surgery training requires several requisite factors to be successful, the most important of which includes exposure to adequate case variety, complexity, and volume. The latter of these has been demonstrated across multiple sub-specialties to be associated with improved surgeon proficiency and, in turn, better patient outcomes. After reviewing the hospital discharge data of 16,954 total thyroidectomy patients, the lowest percentage of complications was recorded with a surgeon performing 21 to 25 cases annually [1]. One-year survival after lung transplantation improved with increasing center volume to as many as 33 cases per year [2]. Acher et al. analyzed the literature (1995–2020) on volume-outcome association in the care of patients with pancreatic cancer. They concluded that hospital/surgeon volume, and patient mortality were inversely related, with the caveat that there could be inherent difficulty in isolating the impact of individual surgeon versus hospital volume [3].

In this commentary, we discuss the benefits and drawbacks of implementing an elective in global surgery from high-income countries (HIC) to low-and-middle income countries (LMIC) to overcome the decline in the total volume of surgical cases with a focus on the open cases.

Several recent events have led to a decline in surgical volume, particularly the number of open cases available for surgical trainees: introduction of duty hour restrictions (DHR), broader use of minimally invasive techniques, and deferral of elective cases due to the COVID-19 pandemic. A survey was linked to the 2020 American Board of Surgery In-Training Examination evaluating residents reported self-efficacy in 10 of the most performed procedures per the Accreditation Council for Graduate Medical Education (ACGME) case logs. Residents reported the highest self-efficacy in wide-local excision (90.24%) while the lowest was in performing open thyroidectomy (19.58%), however, only 7.7% reported self-efficacy in all procedures. Alarmingly, with 5 months left in training, 92.3% of residents reported deficits in preparation for practice [4]. A recent systematic review set out to analyze how the ACGME institution of residency DHR affected resident case volume. The authors noted a decline in surgical case volume at all levels following DHR implementation, most notably in junior residents. To mitigate these effects, some have championed the use of simulation training for the acquisition of technical skills to dampen the learning curve [5].

The deferral of elective surgical care due to COVID-19 has resulted in a huge backlog of numerous elective surgical procedures. It is predicted that the United States may face a cumulative backlog of more than a million total joint and spine surgery cases, while there may be a backlog of 1.1 million to 1.6 million cataract procedures by 2022 [6]. These backlogs will certainly increase volume for future trainees, however, workforce shortages, inpatient bed availability, operating room capacity and physician burnout may negate the increased volume for training [7].

In recent years, global surgery experiences have been adopted by general surgery programs which can serve to expose trainees to surgical problems not routinely seen in the US, while educating them on developing empathic and collaborative approaches to overcome them [8]. The American Surgical Association Working Group for Global Surgery has succinctly summarized the criteria for equitable partnerships by emphasizing the interests of LMIC institutions. There are several proposed benefits of global surgery, including alleviating physician burnout which has been exacerbated during the pandemic [9]. A retrospective analysis of general surgery residents who participated in an international elective demonstrated that 93% experienced a first-time operation, the most common of which included open cholecystectomy and open gynecologic procedures [10]. These findings mirrored the recent global surgery experience of the first author (R.L.) during which he was exposed to a number of first-time operations to include cesarean sections, bladder stone removal, orthopedic fixation, and typhoid bowel perforations, all of which have relevance to general surgery training. This exposure is most relevant for those residents who plan to practice in a rural setting without the ready availability of tertiary subspecialty care, which closely reflects the practices of surgeons in LMIC who typically offer a broader range of procedures. Practicing in more resource-limited settings also imparts valuable lessons in cost-effective surgery, such as suture conservation, and innovative use of surgical tools and instruments [11].

In addition, a structured program in surgical tropical conditions may be beneficial due to increasing immigration and movement of peoples across international borders. Trainees from HIC may also be able to observe first-hand the adverse effects of global warming and climate change, which affects LMIC to a greater extent, and disease processes starting in these areas may become pandemics disrupting surgical services [12].

It can be argued that the idea of using global surgery to compensate for declining surgical volume in the US is inherently inequitable and with little to no benefit to our LMIC colleagues. However, a well-planned collaboration between HIC and LMICs could compensate LMICs at either the institutional level or national level [13]. We propose that funding mechanisms be established for reciprocity so that LMIC trainees should, in turn, be able to travel to HIC institutions for increased clinical exposure to pathology and procedures in which they may have deficits. There should be a formal Memorandum of Understanding between HIC and LMIC, which emphasizes mutual benefits with longitudinal tracking of case logs [14]. There are successful electives such as between the University of Michigan Medical School and institutions in Ghana for over 30 years, which can serve as models [15].

The benefits of global surgery to compensate for decline in volume, variety, and open surgical cases have not been studied robustly, but we believe that residents, and even faculty may be able to increase surgical volume by participating ethically [16] in the national surgical plans of the host country. Potential benefits of global surgery must be balanced with travel costs, cultural differences, language barriers and ethical dilemmas which the trainee may encounter in LMIC, but we believe the time is ripe to study this topic through an academic, ethical, and economic lens.

## Ethical approval

Not applicable.

## Sources of funding

None.

## Author contribution

Laverty and Jindal contributed equally to the study concept or design, data collection, data analysis or interpretation and writing the paper.

## Registration of research studies

Name of the registry: None

Unique Identifying number or registration ID: None

Hyperlink to your specific registration: None

## Guarantor

Dr RM Jindal.

## Consent

Not applicable.

## Disclaimer

The opinions or assertions contained herein are the private ones of the author/speaker and are not to be construed as official or reflecting the views of the Department of Defense, the Uniformed Services University of the Health Sciences or any other agency of the U.S. Government. No financial conflict of interest exists for any of the authors.

## Provenance and peer review

Not commissioned, externally peer reviewed.

## Declaration of competing interest

None.
